# Report of Three Operations of Ovariotomy with Exhibition of Pathological Specimens

**Published:** 1885-02

**Authors:** D. A. K. Steele

**Affiliations:** President Chicago Medical Society, Professor Orthopedic Surgery College Physicians and Surgeons of Chicago, Etc.


### ORIGINAL COMMUNICATIONS.

ARTICLE I.

### REPORT OF THREE OPERATIONS OF OVARIOTOMY WITH EXHIBITION OF PATHOLOGICAL SPECIMENS. *By* D. A. K. STEELE, M. D., *President Chicago Medical Society, Professor Orthopedic Surgery College Physicians and Surgeons of Chicago, Etc.**

*Gentlemen*:—Abdominal surgery is no longer an experimental field. Its brilliant possibilities have become accomplished facts. Doubt and uncertainty have yielded to definite knowledge and precise rules. During the past decade more rapid advancement in operative *technique* with resultant lessened mortality can be claimed in the field of abdominal surgery than in the wider domain of general surgery. To the honored names of Keith, Tait, Thomas, Emmet, and other active workers and writers in this field we desire to add three names from this Society, Jackson, Dudley and Parkes, each of whom by conscientious earnest work has largely aided in Chicago, and the West especially, in advancing and maintaining our knowledge of this subject. And to the latter is due the credit of having written one of the most notable papers presented during the period covered by these preliminary remarks. In presenting reports of three cases of ovarian tumor with operations, I am

* Read before Chicago Medical Society, December 1, 1884.

only adding my mite to the sum total of knowledge in this department, and therefore crave your indulgence while I present:

*Case 1.* Bridget T., aged 59 years, an English washerwoman, was transferred to my care in the Cook County Hospital, June 14, 1880.

Four days previously she was admitted to the Medical Department under the care of Dr. H. P. Merriman, who diagnosed an ovarian cyst. Her family history was interesting from the fact that two older sisters had died of "dropsy," one in the 40th and the other in her 70th year. Father attained an age of 80 years, and her mother died in her 63d year from some lung affection. Mrs. T. did not menstruate until her 20th year. This function continued regularly every four weeks until she was 45, when the menopause occurred without any disturbance of her general health. Her periods had always been painless and lasted two or three days—flow normal in amount.

She was married in her 26th year, but has never been pregnant; enjoyed widowhood for the past 21 years. Has always had excellent health with the exception of an attack of bilious remittent fever about 30 years ago. Was sick a few days "during the war" with "liver complaint," and again about five years ago was in bed a few days with a similar complaint.

In May, 1879, fourteen months prior to her admission to the hospital, she noticed a slight protrusion of the navel, and a few days later noticed a small hard lump as large as a guinea egg in the left illiac region. This seemed hard at times, and again soft; sometimes giving a sensation of heat, and again of cold. This has gradually increased in size until the abdominal cavity was completely filled. Has never experienced any pain in the growth. Frequent micturition with partial incontinence has annoyed her for the past four or five months.

On admission she was fairly nourished; tongue clean, appetite fair, stomach acid, but no nausea or vomiting; bowels regular; pulse 72, respiration 18, tempeaature 98°. *Facies ovariana* fairly marked.

Examination revealed abdomen uniformly enlarged, overlapping ilia and pubes; superficial veins unusually enlarged; girth of abdomen at umbilicus forty-six inches. Manipulation shows enlargement to be firm, resistant and elastic, universally dull and fluctuating, except over an area one and a half inches above navel; line of dullness changes slightly by postural change. No well defined cyst can be outlined by palpation. Vagina is long and narrow; uterus small, atrophic, and drawn upwards. No bulging of Douglas' *cul de sac.* Evidently considerable free ascitic fluid and probably an ovarian cyst.

June 18th, in order to perfect the diagnosis, an attempt was made to draw off the fluid by means of a trocar so as to allow the tumor to be outlined; but unfortunately the cyst was punctured. Thirteen and a half pounds of thick straw-colored fluid were removed; specific gravity, 1011; alkaline reaction, highly charged with albumen, contains Drysdale's cell, cholesterin, epithelium, etc.

Patient was immediately placed in bed, on her back, and kept quiet for three days to prevent fluid escaping from cyst into abdominal cavity. No symptoms followed tapping, and she was around the ward in a few days. The cyst rapidly refilled, and ten days later its firm thick-walled outline, lying chiefly on the right of mesial line, could easily be felt. Lateral motion, on raising it up, transmitted a similar motion to the uterus.

An operation for its removal was decided upon, and she was placed for a week upon preparatory treatment, consisting of a light farinaceous diet, with daily hot baths, frictions and diuretics.

Day before operation she was given milk porridge, a cathartic and hot water vaginal injections, and on the morning of operation an enema of soap-suds.

July 11th, at one P. M., she was etherized for abdominal section. The room in which this operation was to be performed was a private one on the third floor of the west pavilion, and had been thoroughly fumigated, walls washed clean with antiseptic solution and a carbolized spray maintained for two or three hours, temperature brought up to 80°.

The operation was done with the assistance of Drs. C. T. Parkes, Fenger, Jackson, Guerin, McWilliams and the house staff. Some ten or twelve other physicians being present by invitation. The usual incision in the *linea alba* was made, cyst punctured and thick straw-colored fluid drawn off through a Wells trocar; but few adhesions were found, and the cyst which developed from the left ovary was readily drawn out, a temporary fish line ligature applied and the cyst cut off. The right ovary was unaffected—the pedicle was drawn up and tied in three parts by a strong carbolized silk ligature cut short and dropped back. The abdominal cavity was carefully sponged out with fine carbolized sponge to which long strings had been attached in order to guard against the possibility of dropping one—the string was held by an assistant. A good deal of the cystic fluid had escaped into the cavity, and considerable time was occupied in thoroughly sponging it out. A flat sponge was then laid beneath the edges of the abdominal incision, and it was closed by interrupted silk sutures and a complete Lister dressing applied. Patient placed in bed between blankets, surrounded by bottles of hot water and given hypodermic of brandy. Operation lasted nearly two hours. Patient reacted well and made favorable progress for forty-eight hours, when symptoms of pyæmia supervened and death ensued on the fifth day.

A post-mortem examination revealed a small sponge with string attached lying in Douglas' *cul de sac* by the side of stump, and firmly imbedded in lymph. The ligature was embedded in lymph and no hæmorrhage had occurrred. There was a general peritonitis, and one or two pouches containing ill-smelling pus. The cause of death was apparent; but how it was possible to have overlooked the sponge when the final inspection of peritoneal cavity was made, remains a mystery and adds increased weight to the command, "Always count your sponges."

The cyst was a multilocular one, which, with its contents, weighed twenty-nine pounds. I feel certain that but for the accidental presence of a foreign body in the peritoneal cavity, she would have recovered.

*Case II.*—Mrs. Alvina S., aged thirty, a German housewife, was admitted to the Cook County Hospital March 13th, 1882, and gave the following history: Father and mother living and in good health; one sister died at the age of twenty-two from "white janders," excessive anæmia and non-appearance of menses.

Mrs. S. had a healthy childhood; age of puberty at 16; menstruations regularly monthly; duration, three to six days, normal in amount and color, painless. Married at 20.

First child one year later; healthy pregnancy, normal delivery, confined one week. Six weeks after delivery, hæmorrhage, which ceased in four weeks. Regular menstrual flow every two or three weeks since, considerable in quantity; painless; notwithstanding had good health. Dates present illness back to August, 1880, when it began with backache in sacral region, cramping pains from hip to knee occasionally, constipation, throbbing in epigastrium, variable appetite, slept well, no soreness. First noticed tumor in October, 1880, when she began to grow larger around the waist, and supposed she was pregnant until thirteen months had passed. In December, 1880, she noticed a hard, round tumor in the hypogastrium, which increased in size, at times growing rapidly and then remaining stationary. The tumor gradually increased in size until February, 1882, when she was aspirated below umbilicus and ten quarts of a yellowish, greasy-looking fluid were removed, when the tumor entirely disappeared. Four days later she says she again noticed it filling up; sat up in bed then, and fainted for the first time in her life. Remained in bed for three weeks after the tapping. Has emaciated considerable since February.

Examination after admission revealed abdomen enlarged anteriorly, circumscribed bulging with tense walls, universal fluctuation; no partitions into several sacs; no cicatrix of aspirator puncture visible; umbilicus obliterated; palpation shows a moderate sized ovarian cyst, with an apparently harder mass below and to the right side. Vagina normal, uterus small in normal position. No tenderness on examination. March 20th, she complained of burning pains over right side of abdomen; pulse ran up to 106 and temperature to 103.4°. Pulse and temperature continued high for four days, patient complaining of severe pain over abdomen, especially on right side; said it felt as if tumor was going to burst. She was kept under the influence of full doses of opium and a hot poultice applied, when the local peritonitis gradually subsided. March 24th, I aspirated the cysts, removing six quarts of limpid straw-colored fluid relieving dyspnœa and greatly ameliorating most of the distressing symptoms. On the 28th, pulse and temperature were normal, and on April 10th she was able to walk around the ward. On the 14th there was a slight recurrence of the peritonitis. On the 16th I re-examined her carefully and diagnosed an ovarian cyst of left ovary and dedecided upon its removal. April 20th, with the same preparatory treatment and the same antiseptic precautions as in the preceding case, I operated, being assisted by Drs. Gunn, Fenger, Guerin, and the house staff. Patient was etherized at 1 P. M. by Dr. F. S. Johnson. I began the operation twenty-three minutes later, anæsthesia then being complete. The incision in mesial line was five inches in length midway between umbilicus and pubes. Different layers of abdominal wall were divided separately until the cyst wall came into view. Numerous recent slight adhesions were found between the cyst wall and the peritoneum. A large trocar was introduced into the cyst cavity and its contents easily evacuated without any portion of the fluid entering the peritoneal cavity. Pedicle was of medium size, springing from left ovary, and was secured by a temporary ligature of whipcord. Patient was now given 30 drops of brandy hypodermatically. Pedicle was now tied in three sections with No. 10 braided silk ligature and dropped back in abdomen after being cut short. Bleeding from points of adhesion was slight, and was controlled by five cat-gut ligatures. Peritoneal cavity was now thoroughly sponged out and a dry, flat sponge introduced, while external wound was being closed. The primary incision was closed by interrupted silver wire sutures, the flat sponge being withdrawn before the sutures were tightened. A double curved glass drainage tube was introduced at the lower angle of wound, resting in Douglas' *cul-de-sac.* Wound was dressed *a la* Lister and covered with salicylated cotton. Operation lasted one hour and thirty minutes, when patient was placed in bed, given hypodermics of whisky and surrounded by hot rubber-bags, and ordered champagne and ice, catheterized every six hours, and temperature of room to be kept at 75°. Patient reacted well, had considerable vomiting and nausea for the first forty-eight hours, also paroxysmal abdominal pain from movement of intestinal gases, which ceased almost entirely when flatus escaped *per anum* on the third day. On the third day the dressings were removed and changed. The wound was looking well and uniting by first intention. A little odorless serum was drawn out of the glass drainage tube by means of rubber tubing attached to the nozzle of a syringe—was ordered milk and beef tea *per rectum.* On 5th day redressed the wound under spray. No odor and very little discharge about the dressings; wound had united by first intention. Syringe fails to withdraw any fluid from tube. Tube was withdrawn, washed in 3 per cent. carbolized water and replaced. Patients clothing and bed linen also changed. Temperature of room had been gradually brought down to sixty-six. Pulse, temperature and respiration normal and patient permitted to begin taking flour and milk porridge by the mouth. On the seventh day she passed urine naturally for the first time.

Wound was redressed and three of the silver wire sutures removed, and the drainage tube left out permanently. On the tenth day all sutures were removed and the sinus left by drainage tube was found to be rapidly healing. On the eleventh day the bowels moved after an enema quite freely. May 4th the patient sat up in a chair for forty minutes without feeling fatigued. May 12th the wound had entirely healed and a nicely fitting abdominal bandage was applied to support the relaxed abdominal wall.

*Summary.*—Duration of tumor, since August, 1880, (21 months.) Tapped February 5th, 1882, ten quarts fluid withdrawn. Tapped March 25th, 1882, six quarts fluid withdrawn.

Operation April 20th. Weight of tumor 19 pounds. Highest temperature following, 100.2 (3d day). Temperature continued normal after fourth day. Sat up on 14th day. Discharged recovered on 24th day. Eleven days after she left the hospital her left leg became painful and swollen to twice its natural size by reason of pressure on the illiac vein from plastic deposit around the pedicle. This yielded entirely in a few weeks to rest and bandaging, and she has been perfectly well ever since, menstruating regularly.

*Case 3.*—Mrs. A. L. M., aged 39 years, a widow, born and always resided in Chicago, mother of seven children, three living, came under my observation September 22d, 1884. I first saw her at my office and elicited the following history: puberty was established at the age of 13, and the flow was always profuse and accompanied with pain. She was married at the age of 17, and conceived four months afterwards. Carrying the child to the full period of gestation, being perfectly healthy. Her labor was very difficult, the child being born double. She was in bed three weeks after her confinement, at the close of which time she sat up, and at the expiration of a month began to go about the house, but was not fully up to her normal condition until eleven months afterward. In one year and eight months after the birth of her first child, she conceived again and carried the child but seven months when she was delivered of a boy weighing three pounds. Child lived eighteen months. Patient thinks she would have carried the child the full term of gestation had it not been for a shock to her system brought on by the death of her mother. She conceived again one year after the birth of her second child (not having had a return of the menses), and a fourth child was born two years afterwards without any indications of the menstrual flow. When nearly five months advanced with her fifth child she had a discharge simulating the menstrual flow but lasting only one day, after which she saw nothing to indicate a return of the catamenia until after the sixth child was born, in February, 1874. When the sixth child was sixteen months old the menses returned and continued regularly and without pain until she conceived the seventh and last time, October, 1878. After recovering from the last confinement, patient was well until July, 1879, when she experienced severe pain in the left hypochondrium so severe in character as to cause her to consult a physician. All measures taken to secure permanent relief proved futile—the patient growing thinner and weaker until two years ago, when she noticed an unusual fullness of the abdomen just before each menstrual period, and which became less noticeable after the flow subsided. One year ago, when bathing one day, she discovered a swelling in the right side, the dimensions of which were like a large saucer, and which steadily increased in size, giving the patient much discomfort, thus inducing her to again consult a physician, which she did, as previously stated, Sept. 22nd, 1884. She presented at this time a markedly cachectic emaciated appearance, abdomen protruded anteriorly. Prominence was circumscribed to the right of mesial line chiefly, dull on percussion, had a knobby projection and extended down into pelvis, fluctuation being marked. Uterus and vagina normal. Diagnosed a probable multilocular ovarian cyst.

Sept. 24th, two days afterwards, Drs. Jackson and Dickerson saw her, in consultation with me, and confirmed the diagnosis. An operation was advised, and on Nov. 13th the patient entered Dr. Dickerson's private hospital and took preparatory treatment for it. (Hot baths, diuretic, etc.)

Sunday, Nov. 16th, I operated upon her, with the assistance of Drs. Jackson and St. John, and in the presence of Drs.

Earle, Martin, Doering, Byford, Beery, and Messrs. Barlow, Casely and Earle, medical students.

Dr. Dickerson administered the anæsthetic (ether). The temperature of the room was kept at 80°, the air was saturated with carbolized vapor, and the usual precautions of absolute cleanliness observed. At 2:12 the first incision was made, and at 2:57 she was placed in bed, the operation having been fully completed. The primary incision in linea alba was about three inches long, midway between umbilicus and pubes. The tissues were so anæmic that there was no need of using hemostatic forceps, all bleeding points ceased spontaneously a few minutes after the pearly bluish white cyst wall came into view. The cyst was emptied by an Emmet's trocar with tubing attached. No adhesions were found, and the cyst involved the right ovary. The fluid was a dark amber. A temporary ligature of hemp twine was thrown around the pedicle, which was now divided. A cyst of the left ovary, about the size of a fœtal head, now came into view and was emptied by the trocar of ten or twelve ounces of thick, yellow, purulent-looking fluid. This pedicle was also secured by a temporary ligature and cut off. Both pedicles were now secured and tied in two parts by heavy braided silk (No. XI), cut short and dropped back in the abdomen. The peritoneal cavity was now carefully sponged out and rinsed two or three times with warm carbolized water (one per cent.) and sponged dry. No oozing occurred, and no fluid from the cyst was permitted to enter the abdominal cavity. A warm, flat carbolized sponge was now placed beneath the primary incision to absorb any blood that might escape from the needle punctures. The wound was closed by nine interrupted silk sutures and an iodoform dressing applied, covered with salicylated cotton held in place by fine strips of rubber adhesive plaster, and over all a firm flannel binder. Patient's knees were flexed over a firm pillow upon the bed, and after recovering from under the influence of the anæsthetic her pulse was 96; temperature, 98½°; respiration, 22. At no time afterwards did her temperature rise above 100°, or her pulse over 94. She had no vomiting, and absolutely no pain; slept considerable. She was nourished on milk, ice and whisky for the first week, then on milk, porridge, beef tea and animal broth afterwards, no solid food being administered for ten days. No drugs were given. She made a steady rapid recovery without any symptoms to record. Catheter was used every six hours for five days; after that she urinated naturally. Flatus escaped *per rectum* on the second day; bowels were moved by enema on the eleventh day. The dressing was not disturbed until the seventh day, when union was found to be perfect by primary adhesion. All the sutures were removed, the line of union was powdered with iodoform, and a fresh dressing applied. This remained undisturbed until she was able to sit up; occasionally blew a little iodoform under the edges of the dressing by means of an insect powder bellows.

*Summary.*—Duration of tumor (probably since 1879) certainly since 1883.

Sept. 22d, 1884, ovarian cyst diagnosed.

Nov. 9th, 1884, two ovarian cysts removed; weight, nine pounds.

Highest temperature after operation, 100° (third day.)

She sat up on the thirteenth day, and was discharged recovered on the nineteenth day after the operation. Examination of cysts showed them to be filled with numerous papilliform growths.

The conclusions to be deduced from a clinical study of these three cases may be summarized thusly:

(*a*) Always count your sponges.

(*b*) Absolute cleanliness is more important than absolute Listerism.

(*c*) Peritonitis coëxisting is not a contra-indication for either tapping or operation.

(*d*) Cystic fluid left in peritoneal cavity is dangerous and likely to cause pyæmia; blood is probably innocuous.

(*e*) A rubber blanket with a fenestrum cut in its center through which to operate, and fastened to the belly by a circular adhesive, is useless to prevent the cystic fluid from running over the patient and operating table, as the plaster is usually accidentally pulled off by turning the patient over during a fit of vomiting, or by some over-zealous spectator who seeks to guide the flow of fluid from its folds to the tub or receptacle; better far omit it, and use small tin basins that can be readily changed as needed.

(*f*) Each case calls for special judgment and individual attention to every detail.

